# Circadian rhythms of melatonin and its relationship with anhedonia in patients with mood disorders: a cross-sectional study

**DOI:** 10.1186/s12888-024-05606-5

**Published:** 2024-02-27

**Authors:** Xinyu Li, Jiakuai Yu, Shuo Jiang, Liang Fang, Yifei Li, Shuangshuang Ma, Hui Kong, Ximing Qin, Daomin Zhu

**Affiliations:** 1 The School of Mental Health and Psychological Sciences, Anhui Medical University, 230032 Hefei, China; 2 Department of Sleep Disorders, Anhui Mental Health Center, 230022 Hefei, China; 3 Institutes of Physical Science and Information Technology, Anhui University, 230039 Hefei, China; 4 Department of Sleep Disorders, Hefei Fourth People’s Hospital, 230022 Hefei, China; 5 School of Nursing, Anhui Medical University, 230032 Hefei, China

**Keywords:** Depression, Bipolar disorder, Anhedonia, Circadian rhythm, Melatonin, Mood disorder

## Abstract

**Background:**

Mood disorders are strongly associated with melatonin disturbances. However, it is unclear whether there is a difference in melatonin concentrations and melatonin circadian rhythm profiles between depression and bipolar disorder. In addition, the relationship between anhedonia, a common symptom of affective disorders, and its melatonin circadian rhythm remains under-investigated.

**Methods:**

Thirty-four patients with depression disorder, 20 patients diagnosed with bipolar disorder and 21 healthy controls participated in this study. The Revised Physical Anhedonia Scale (RPAS) was performed to assess anhedonia. Saliva samples were collected from all subjects at fixed time points (a total of 14 points) in two consecutive days for measuring the melatonin concentrations to fit circadian rhythms of subjects. Melatonin circadian rhythms were compared between the three groups using ANOVA. Partial correlation analysis and linear regression analysis were used to explore the correlation between melatonin rhythm variables and anhedonia.

**Results:**

We found that the peak phase of melatonin in the depression group was significantly advanced compared to the control group (*P* < 0.001) and the bipolar disorder group (*P* = 0.004). The peak phase of melatonin and RPAS showed a negative correlation (*P* = 0.003) in depression patients, which was also demonstrated in the multiple linear regression model (B=-2.47, *P* = 0.006).

**Conclusions:**

These results suggest that circadian rhythms of melatonin are differentiated in depression and bipolar disorder and correlate with anhedonia in depression. Future research needs to explore the neurobiological mechanisms linking anhedonia and melatonin circadian rhythms in depressed patients.

## Introduction

Mood disorders are one of the most disabling diseases in the world, with a significant burden on both individuals and society [1]. Mood disorders include depressive disorder (DP) and bipolar disorder (BD), but it is difficult to distinguish the depressive episodes of BD from those of DP in a clinical setting, particularly in the initial stage of both disorders. People with bipolar disorder are therefore often misdiagnosed with DP. It is reported that nearly 40% of BD patients are initially misdiagnosed with DP [2, 3], resulting in poor prognosis for many bipolar patients. Therefore, it is necessary to find objective clinical bioindicators that can assist in distinguishing depression disorder from bipolar disorder.

Over the past 50 years, a growing body of research has shown that circadian disruptions are associated with mood disorders. As research on the circadian system has progressed, the cyclic rise and fall of melatonin have been used as markers of the circadian phase for measuring the effects of light exposure [4]. Evidence is accumulating on a possible role for melatonin in influencing mood [5, 6], and melatonin-related drugs have been an effective option for individuals with mood disorders comorbid with circadian rhythm disruption [7, 8]. Significantly lower melatonin levels were found in bipolar disorder patients during manic, and depressed phases compared to healthy individuals, even in remission patients [9].A prospective cohort suggested circadian rhythm regularity significantly predicted the time onset of depressive episode of bipolar disorder [10]. More studies have tested melatonin as a treatment strategy for depressive episodes in mood disturbance [11, 12]. Thus, abnormal circadian amplitude and disturbed timing of melatonin rhythms have been observed in individuals with depression disorder and bipolar disorder. However, the differences in melatonin secretion and its circadian rhythm changes between BD and DP are not clear, which needs to be clarified.

Anhedonia, loss of interest or pleasure in usual activities, is a characteristic of depression and some types of anxiety, as well as substance abuse and schizophrenia [13]. Anhedonia has been a predictor of poor long-term outcomes of affective disorders, including suicide and poor response to treatment. Because extant psychological and pharmacological treatments are relatively ineffective for anhedonia, there is an unmet therapeutic need. Ample evidence supported that DP and BD patients have more common and/or severe anhedonia compared to the healthy controls [14, 15]. In clinical practice, studies have shown agomelatine has some specific effects on the symptoms of anhedonia in DP, but it has rarely been reported in BD [16–18]. However, that agomelatine differentially affects DP but not BD is not well understood. Furthermore, an animal model found that deletion of MT2 receptors, a principal type of G protein-coupled receptor that mainly mediates the effects of melatonin, in rodents impairs regulation of reward sensitivity, resulting in anhedonia [19]. Therefore, it remains to be confirmed whether melatonin rhythmic oscillation may be involved in anhedonia in patients with mood disorders.

Based on our previous research finding that melatonin rhythms could characterize the different clinical phases of bipolar disorder [20], we hypothesized that the circadian profile of melatonin may differ in BD and DP. Here, we conducted a case-control study to investigate differences in melatonin concentrations assessed from salivary samples in DP patients, BD patients and healthy individuals. We also explored the association between melatonin changes and anhedonia in people with depression.

## Materials and methods

### Participants

Patients with DP, BD were recruited at the department of sleep disorders, psychological hospital affiliated to Anhui Medical University, China, and healthy controls (HC) were recruited from the community. From March 2019 to April 2022. A total of 75 participants (DP group, *n* = 34; BD group, *n* = 20; HC group, *n* = 21) were enrolled in the study. All participants were aged 16 to 65 years old, and had a primary school and above education. All participants with depressive disorders were assessed by two mental health professionals using DSM-IV criteria to determine all psychiatric diagnoses [21]. Participants were excluded from the study if they had a history of neurological disorders or head injuries, or other major psychiatric disorders; or if they had received electroconvulsive therapy or transcranial magnetic stimulation within 3 months; or if they had a serious physical illness that might interfere with the study evaluation; or they were currently using hydrocortisone or prednisolone. Additionally, individuals with a lifetime personal or immediate family history of mental illness were excluded from the control group.

The study protocol was approved by the Institutional Review Committee of Psychological Hospital Affiliated to Anhui Medical University (HFSY-IRB-PJ-ZDM). Written informed consent was obtained from all participants or their legal guardians before enrollment.

### Procedures

**Fig. 1:**
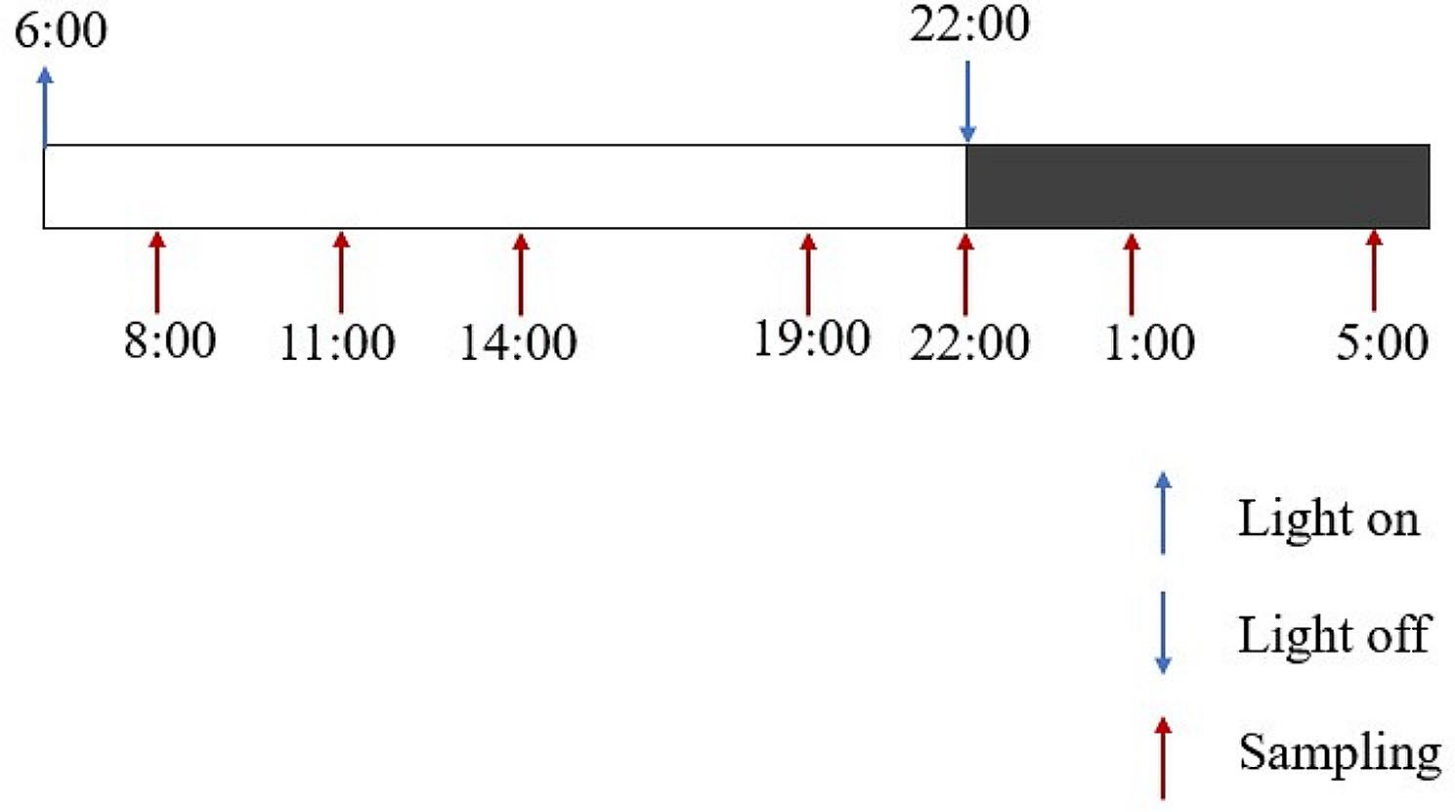
Schematic of the protocol design. The figure illustrates the protocol used in this study. Our sleep monitoring laboratories lights up at 6:00 (indicated by the blue upward arrow) and lights off at 22:00 (indicated by the blue downward arrow). In the laboratory, subjects can maintain their normal activity during waking time. Subjects need to stay in the laboratory for three days. They spend the first day acclimatizing to the laboratory environment without being sampled. Samples are taken at indicated time points (08:00, 11:00, 14:00, 19:00, 22:00, 01:00 and 05:00) on the following two consecutive days. Light levels were maintained at about 400 lx during the light-on period and less than 20 lx during the light-off period

The study protocol is depicted in Fig. 1. Participants were required to go to bed at 22:00 and wake up at 06:00. Light exposure was also controlled on a daily basis by the ward routine. The lights were turned on from 06:00 to 22:00 (light intensity is about 400 lx) and turned off from 22:00 to 06:00 (light intensity does not exceed 20 lx). The ward curtains were closed, so that all subjects avoided exposure to natural daylight. Before sampling, all participants were required to keep regular sleep-wake schedule for the past week. Additionally, all subjects needed one day to adapt to the ward, and saliva samples were taken by well-trained nurses on the second and third days. Subsequently, their saliva was sampled at 08:00, 11:00, 14:00, 19:00, 22:00, 01:00 and 05:00 o’clock for two consecutive days, respectively (Fig. 1). The saliva (≥ 1 ml) was sampled into a sample tubing (Salivette®, Sarstedt AG & Co, Numbrecht, Germany), and stored at − 40℃ until measurements. During the sampling, participants were asked not to drink any caffeinated or alcoholic beverages. They were also asked to refrain from cleaning their teeth or chewing gum for at least 15 min before each sampling time. The nighttime sample collection was performed by a well-trained experimental staff in the dimmest possible light. The collection did require brief awakenings, and it is known that patients with acute psychiatric disorders often have disturbed sleep.

### Clinical assessments

The Revised Physical Anhedonia Scale (RPAS) was developed by Chapman in 1978 and translated by linguists and psychologists into a Chinese version of the RPAS [22]. The RPAS is a measure of stable individual differences in the ability to experience pleasure from physical-sensual, such as food, sex, and settings.The RPAS contains 61 true-false items. These items are scored relative to the standard answers. An answer to a question is scored as “1” if it matches the standard answer to that question; otherwise, it is scored as “0”. Higher scores are indicative of more severe anhedonia. Both the original English RPAS (α = 0.74) and the Chinese RPAS (Cronbach’s α = 0.75) have good internal consistencies [23].

The Hamilton Depression Scale (HAMD) and the Hamilton Anxiety Scale (HAMA) are widely used to assess the appearance of depression and anxiety, respectively. HAMD contains 24 items [24], and HAMA contains 14 items [25]. The scale is scored as 0 (never), 1 (mild), 2 (moderate), 3 (severe), and 4 (extremely serious). In HAMD, the total score ranges from 0 to 8 for no depression, 21 to 35 for mild to moderate depression and ≥ 36 for severe depression. In HAMA, the total score ranges from 0 to 56, with higher scores indicating more severe depressive symptoms. Various of previous studies have shown that these questionnaires could assess psychological condition with satisfactory reliability and validity [24, 25].

#### Salivary melatonin measurement

Salivary melatonin was measured using Melatonin Direct Saliva ELISA (IBL International GmbH, Hamburg, Germany), following the product instructions. The limit of quantitation was 0.854pg/ml (coefficient of variation = 20%). The lntra assay precision showed a mean coefficient of variation for day samples (< 8 pg/mL) of 17.0% (15.0–19.0%) and for night samples (> 10pg/mL) of 13.9% (13.2–14.4%). The Inter assay precision showed a mean coefficient of variation for day samples (< 8 pg/mL) of 20.5% (17.1–23.8%) and for night samples (> 10pg/mL) of 18.4% (16.5–19.7%). The mean between lot variation was 13.4% (8.8–17.7%). Melatonin concentrations are expressed as pg/ml at each time point.

### Statistical analysis

One-way ANOVA and Welch’s ANOVA were used to assess differences between the three groups (BD, DP and HC) in age, education, physical activity, RPAS, HAMD, HAMA, and melatonin variables. A two-sample t-test was performed to compare general demographics and melatonin concentrations between the DP and BD groups. All continuous variables results are shown using the mean (standard error of mean, SEM). Chi-squared test was performed on the categorical variables (for gender only). In addition, we used a partial correlation analysis to test the association on the peak phases of the salivary melatonin across diagnostic groups, adjusted for age, gender, education, and physical activity [26, 27]. Multiple linear regressions were also run to investigate the association between melatonin peak phase and RPAS scores in the DP group. Analyses were deemed statistically significant at *P* value < 0.05.

Melatonin data for each person were nonlinearly fitted with the definition of *Y* = *mesor*+ [*amplitude* * cos (2π * (*X*– *acrophase*)/*wavelength*)], with wavelength invariant over 24 h. GraphPad Prism was used to achieve nonlinear curve fitting, and the mesor, amplitude, acrophase of the curve, and coefficient of determination R2 (goodness of fit) of the curve were calculated. Then the circadian rhythm variables of each person’s saliva melatonin were extracted separately, such as wavelength, acrophase, fitted peak value, and fitted mesor. Using melatonin concentrations from two consecutive days, we calculated the area under the curve (AUC) of mean daily salivary melatonin over time [28].

All statistical analyses were performed using SPSS (IBM SPSS Statistics, version 25, Chicago, IL) and plotting was performed in GraphPad Prism (version 9.5, La Jolla, CA).

## Results

### Descriptive characteristics of samples

The sample characteristics are showed in Table 1. This study included 20 bipolar disorder (BD) patients (3 male and 17 female), 34 depressive disorder (DP) patients (10 male and 24 female), and 21 healthy subjects (7 male and 14 female). The mean age (SEM) of the participants were 31.45 (2.96) years old for BD, 33.26 (2.07) years old for DP and 32.05 (2.89) years old for HC, respectively. There were no significant differences with respect to demographic data including age, sex, education and physical activity among the three groups (*P* = 0.868; *P* = 0.368; *P* = 0.219; *P* = 0.849). In terms of clinical variables, there were significant differences between the three groups in RPAS, HAMD and HAMA scores (*P* < 0.001; *P* < 0.001; *P* < 0.001). The RPAS of the BD patients was 25.20 (3.00), and that of the DP patients was 25.68 (2.19). The RPAS scores of BD and DP were not significantly different (*P* = 0.897). Similarly, no significant differences in HAMD and HAMA were found in the comparisons between the BD group and the DP group (*P* = 0.109; *P* = 0.523).

**Table 1:** Demographic characteristics and clinical variables of the recruited subjects

Variables	HC (*n* = 21)	BD (*n* = 20)	DP (*n* = 34)	*P* value	BD vs. DP ^a^
Age, years, mean (SEM)	32.05(2.89)	31.45(2.96)	33.26(2.07)	0.868	0.609
Sex, Female, n (%)	14(66.67)	17(85.00)	24(70.59)	0.368	0.232
Education, years, mean (SEM)	13.95(0.70)	12.15(1.03)	12.24(0.64)	0.219	0.941
Physical activity^b^, times/week, mean (SEM)	2.29(0.23)	2.32(0.31)	2.15(0.19)	0.849	0.623
RPAS scores, mean (SEM)	8.19(1.05)	25.20(3.00)	25.68(2.19)	< 0.001	0.897
HAMD scores, mean (SEM)	1.33(1.96)	31.85(1.53)	28.71(1.17)	< 0.001	0.109
HAMA scores, mean (SEM)	0.67(1.28)	18.25(1.28)	19.29(0.99)	< 0.001	0.523

^a^*P* value analyzed with independent sample T-test were used for comparison between group BD and group DP

^b^ Physical activity indicates the number of days per week with at least 10 min of physical activity.

HC = healthy control; BD = bipolar disorder; DP = depressive disorder; RPAS = Revised Physical Anhedonia Scale; HAMD = Hamilton Anxiety Scale; HAMA = Hamilton Depression Scale

### Changes of salivary melatonin concentrations in the bipolar disorder, depressive disorder and Control Group

**Fig. 2:**
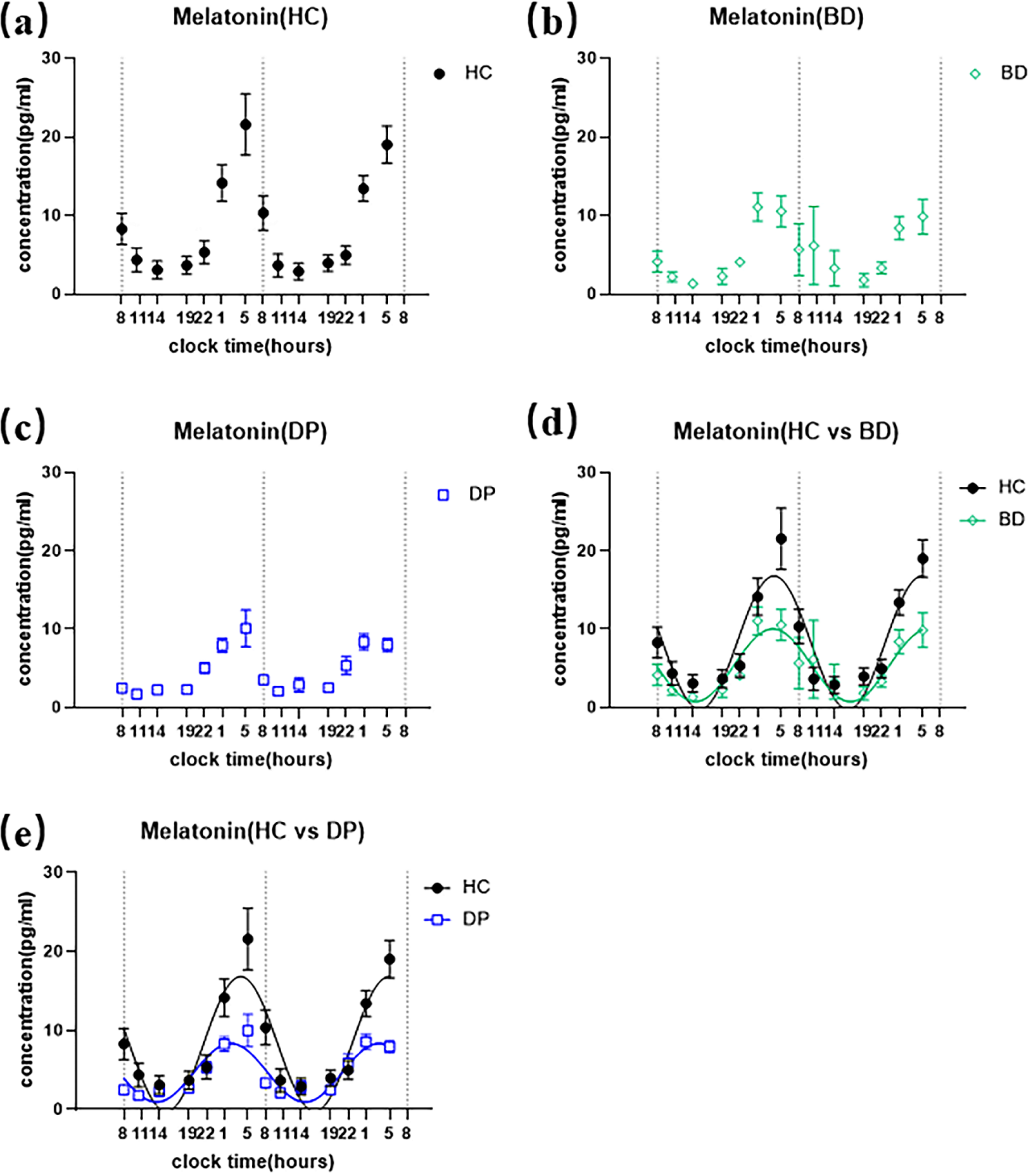
Two consecutive days profile of melatonin concentrations in collected saliva samples from recruited subjects. X-axis shows the sampling time points at the clock time. (a) Averaged melatonin measurement from healthy controls (n = 21). (b) Averaged melatonin measurement from BD patients at depression episode (n = 20). (c) Averaged melatonin measurement from DP patients (n = 34). (d) Cosine curves were used to fit the measured melatonin values from both healthy controls and depressive BD patients. The dashed line presents the fitted curve of healthy controls, and the solid green line presents the fitted curve of depressive BD patients. (e) Cosine curves were used to fit the measured melatonin values from both healthy controls and DP patients. The solid blue line presents the fitted curve of recovery DP patients

The melatonin profiles of all subjects, including recruited BD patients, DP patients and healthy controls, showed robust circadian rhythms when the melatonin contents in collected saliva were measured. A cosine analysis exhibited significant conforms to each individual. Averaged data of the melatonin profiles were showed as three groups: healthy controls (Fig. 2a), BD patients (Fig. 2b), and DP patients (Fig. 2c). In addition, we compared the melatonin profiles in HC versus BD (Fig. 2d), and HC versus DP (Fig. 2e).

### Comparison of melatonin circadian rhythm between control group and patient groups

**Fig. 3:**
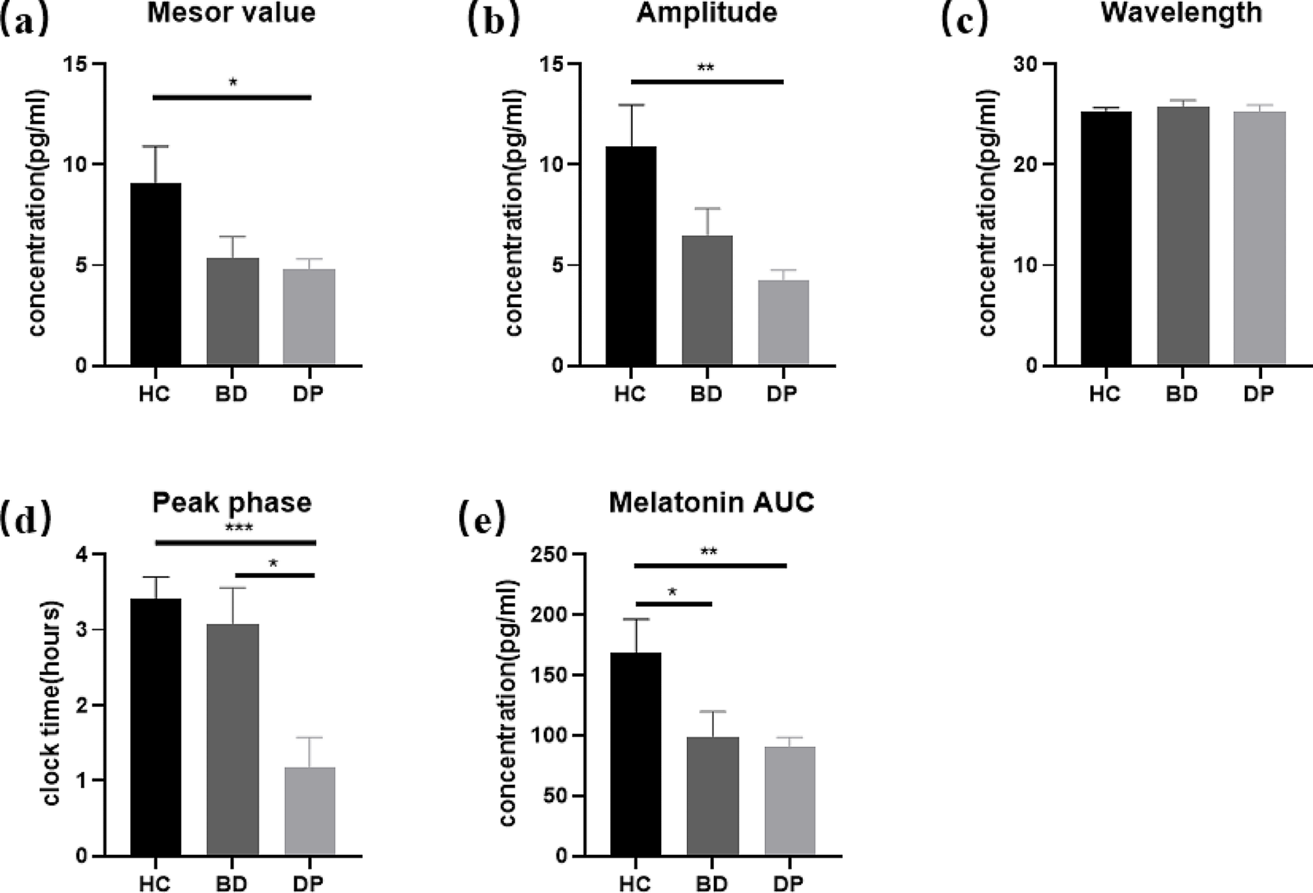
Analysis of various parameters of saliva melatonin secretion in two consecutive days from recruited subjects (mean ± SEM). **(a)** Mesor value of the melatonin rhythm, calculated by the fitted cosine curve in Fig. 2. **(b)** The amplitude of melatonin in the fitted curves for each group. **(c)** The wavelength of melatonin in the fitted curves for each group. **(d)** The peak phase of melatonin in the fitted curves for each group. **(e)** The average area under the curve for two consecutive days, calculated based on the salivary melatonin concentration. Significance levels are displayed as *P* < 0.05 (^*^), *P* < 0.01 (^**^), and *P* < 0.001 (^***^)

According to the averaged melatonin values at 14 sample time points on two days in each group, we calculated various parameters of the salivary melatonin (Fig. 3). In BD group the mesor value and the amplitude of melatonin were 5.34 (1.07) pg/ml and 6.51 (1.31) pg/ml. Besides, those variables in DP group were 4.82 (0.48) pg/ml and 4.22 (0.53) pg/ml. Significantly decreased mesor value and decreased amplitude were observed in DP groups, compared with healthy controls (Fig. 3a, *P* = 0.040; Fig. 3b, *P* = 0.005). The wavelengths of melatonin in the BD and DP groups were 25.74 (0.68) pg/ml and 25.31 (0.61), respectively, which were not significantly different from the control group (Fig. 3c, *P* = 0.603, *P* = 0.953). After fitting the melatonin profile with cosine curves, the peak phases showed significant differences between DP (1.18(0.39) pg/ml) and HC (3.42(0.29)) (Fig. 3d, P = *P* < 0.001). The mean AUC of the salivary melatonin concentrations were significantly lower in BD (99.10(20.49) pg/ml) and DP group (90.79(7.73) pg/ml) than in control group (Fig. 3e, *P* = 0.037, *P* = 0.005).

### Comparison of melatonin circadian rhythm between bipolar disorder and depressive disorder groups

We compared the circadian rhythms of melatonin between BD and DP groups (Table 2). The mesor values of melatonin between two groups were not different, nor were the amplitude (*P* = 0.616; *P* = 0.117). Similarly, no differences in the wavelength of melatonin and melatonin AUC were observed between BD and DP groups (*P* = 0.655; *P* = 0.652). However, as compared to the BD group whose peak phase was 3.08 (0.48) pg/ml, the peak phase of DP group with 1.18 (0.39) pg/ml was significantly advanced (*P* = 0.004).

**Table 2:** Comparison in circadian rhythms of melatonin between BD group and DP group

Parameters	BD	DP	*t* value	*P* value
Mesor value, pg/ml, mean (SEM)	5.34(1.07)	4.82 (0.48)	-0.504	0.616
Amplitude, pg/ml, mean (SEM)	6.51(1.31)	4.21(0.53)	-1.624	0.117
Wavelength, pg/ml, mean (SEM)	25.74(0.68)	25.31(0.61)	-0.450	0.655
Peak phase, hours, mean (SEM)	3.08(0.48)	1.18(0.39)	-3.015	**0.004**
Melatonin AUC, pg/ml, mean (SEM)	99.10(20.49)	90.79(7.73)	-0.454	0.652

### Association between circadian rhythm and anhedonia in mood disorders

In the relationship between melatonin circadian rhythm and clinical symptoms, after adjusting for demographic and clinical variables, the peak phase of depressive patients was negatively correlated with RPAS in partial correlation analysis (*P* = 0.003), indicating that the peak phase was associated with RPAS in depression patients. We found that the peak phase was strongly negatively associated with RPAS in the model (Crude model: B=-2.74(-4.49,-0.99), *P* = 0.003). Further, similar association was detected in the model that was additionally controlled for age, gender, education, and physical activity (Adjusted model: B=-2.47(-4.29,-0.65), *P* = 0.006). Linear regression between RPAS scores and melatonin circadian rhythm in mood disorder was showed in Table 3. No link has been found between melatonin rhythm and anhedonia in patients with bipolar disorder.

**Table 3:** Association of melatonin parameters with RPAS scores by DP patients and BD patients

Melatonin parameters	RPAS Scores				
	BP group, B (95% CI)			DP group, B (95% CI)	
	Model 1	Model 2		Model 1	Model 2
Mesor value, pg/ml	-0.45(-0.91,1.82)	0.33(-1.18,1.83)		0.33(-1.30,1.95)	0.75 (-0.95,2.45)
Amplitude, pg/ml	0.18(-0.96,1.31)	0.01(-1.23,1.24)		-0.11(-1.59,1.37)	0.15(-1.34,1.63)
Wavelength, pg/ml	0.40(-1.79,2.59)	0.89(-1.44,3.22)		0.59(-0.68,1.86)	0.12(-1.23,1.47)
Peak phase, hours	1.67(-1.32,4.66)	1.90(-0.11,0.06)		-2.74(-4.49,-0.99) ^**^	-2.47(-4.29,-0.65) ^**^
Melatonin AUC, pg/ml	0.04(-0.05,0.12)	0.02(-0.08,0.11)		-0.02(-0.13,0.10)	0.03(-0.10,0.17)

Model 1: Unadjusted confounding factor;

Model 2: Adjusted for age, sex, education and physical activity

^**^ Indicates *P* < 0.01

## Discussion

The findings of this study support that melatonin secretions and its circadian rhythm profile are associated with mood disorders. We found a 2-hour phase advance of melatonin rhythms in the DP patients, while BD patients showed no significant difference compared to controls. The mesor value and amplitude of melatonin were significantly damped in the DP patients compared to the HC, while there was no significant difference in BD patients. In addition, a correlation that the earlier peak phase, the more severe the anhedonia in DP was found. Previous studies have also suggested that misalignment of circadian rhythms is a possible mechanism for mood disorders [29, 30]. In our results, DP patients have been associated with markedly lower amplitude of melatonin compared to healthy controls, consistent with the findings of most studies [31, 32]. There is evidence that low amplitudes of melatonin make circadian clocks more susceptible to perturbations in the external environment [33]. This is consistent with the suggestion that reduced melatonin secretion around bedtime could potentially contribute to sleep onset difficulties. However, in our study, there was no difference in amplitude of melatonin between HC and patients with BD in the depressive phase.

### The peak phase of melatonin is advanced in patients with depression and bipolar depression compared to healthy controls

The current results suggest that the melatonin peak is advanced in depression patients. While some of the first studies assessing melatonin in patients with mood disorders found a phase-delay of melatonin onset or peak [34, 35], several others reported an advance in the peak or onset of melatonin secretion [36, 37]. The differences between these studies may be due to differences in melatonin sampling method or to factors related to light and season. In our study, we used a non-invasive method to collect saliva and obtained relatively stable peak phase by fitting the cosine curves of melatonin concentrations at 14 time points (Fig. 2). In addition, we strictly controlled the timing and intensity of the light to reduce the effect of other factors on the circadian rhythm. Besides, after expanding the sample size, our results were consistent with the results of our previous research, which indicated the stability of the results [20].

### The peak phase of melatonin is advanced in patients with unipolar depression compared to bipolar depression

Compared with bipolar depression, the peak of melatonin in unipolar depression was earlier in this study, consistent with the findings of most studies [35, 38]. Interesting, the finding of advanced circadian phase associated with sleep is consistent with features of unipolar depression, such as early morning awakenings [39], difficulty falling asleep and shorter rapid eye movement (REM) sleep onset latency [40]. These features are suggestive of a relative circadian phase advance propensity in depression. However, Weglarz et al. found no differences in serum melatonin rhythm observed between depressive disorder and bipolar depression [41]. The different results may be due to the different ways in which the melatonin peak phase is measured. In the study of Weglarz et al., the peak phase was estimated from the collected blood melatonin concentrations, while in our study, a relatively more stable peak phase obtained from a cosine curve fit to the melatonin concentration. Our results suggest that there are differences in the peak phases of melatonin between bipolar disorder and depression, and future studies with larger sample sizes are needed to support this idea.

### The more advanced the peak phase of melatonin in patients with unipolar depression, the more severe the anhedonia

Interestingly, our observations indicate a strong correlation between the peak phase of melatonin and anhedonia in DP patients.This is similar to previous animal results showing that depression model rats showed anhedonia after rhythm disturbance [19, 42, 43]. Actually, anhedonia in depression is related to the dysfunction of the reward circuit, a neural network with a dense distribution of dopamine [44]. Ample studies have consistently demonstrated structural and functional aberrance in reward system across patients with BD and DP [45–47]. These findings suggest that the neural mechanisms underlying the anhedonia in BD and DP may be distinct. This may be why we did not find a correlation between melatonin phase and anhedonia in bipolar depression. In addition, an animal study showed that depressed mice treated with melatonin enhanced expression of dopamine-related proteins [48]. Agomelatine, a melatonin agonist, exhibits indirectly dopaminergic properties [49, 50]. Thus, we can speculate that melatonin may affect anhedonia by affecting the dopaminergic system. Further experiments will be necessary to explore the mechanisms between circadian rhythm and anhedonia in mood disorders and whether anhedonia symptoms in depressed patients can be treated by modulating melatonin rhythms.

### Strengths and limitations

Our findings provide new evidence for changes in melatonin rhythms in patients with unipolar and bipolar depression. The peak phase of melatonin may be a potential biological feature of depression. Also, our results suggest that better understanding the impact of melatonin rhythms on anhedonia in the future may provide avenues for therapies to improve anhedonia in depression. There are several limitations in the current study. First, for ethical reasons, we recruited patients who inevitably used drugs during their hospitalization. However, we excluded patients using drugs that have an effect on melatonin secretion (e.g., agomelatine, fluvoxamine) at the time of recruitment. Second, lack of data on light exposure for a week or more prior to admission. Because we included patients who were already hospitalized, we were unable to obtain data on patients prior to hospitalization. Third, we assessed anhedonia using self-report. Anhedonia arises from dysfunctional interactions between stress and brain reward systems that may be influenced by the ability to self-regulate [51]. Specific assessment techniques need to be optimized in the future to further investigate the biological mechanisms of anhedonia in depression. More studies are urgently needed to explore whether melatonin can regulate rhythm and improve anhedonia at the same time.

## Conclusions

Melatonin secretion and its circadian rhythm oscillation might be recognized as a strong clinical reference index to assist in differentiating between unipolar depression and bipolar depression, and that as significantly associated with anhedonia in patients with depression.

## Data Availability

The datasets generated and analyzed during the current study are not publicly available to protect participants’ privacy but are available from the corresponding author on reasonable request.

## List of abbreviations

AUC: Area Under the Curve
BD: Bipolar Disorder
DP: Depressive Disorder
DSM-IV: Diagnostic and Statistical Manual of Mental Disorders-IV
HAMD: Hamilton Depression Scale
HAMA: Hamilton Anxiety Scale
HC: Healthy Controls
RPAS: Revised Physical Anhedonia Scale
